# The role of the public and private health sectors on factors associated with early essential newborn care practices among institutional deliveries in Ghana

**DOI:** 10.1186/s12913-021-06665-0

**Published:** 2021-06-30

**Authors:** Maxwell Tii Kumbeni, Paschal Awingura Apanga, Mary-Ann Wepiamo Chanase, John Ndebugri Alem, Nana Mireku-Gyimah

**Affiliations:** 1 Ghana Health Service, Nabdam District Health Directorate, Nangodi, Ghana; 2 School of Community Health Sciences, University of Nevada, Reno, USA; 3 College of Applied Health Science, University of Illinois, Urbana-Champaign, USA; 4 School of Nursing and Midwifery, University for Development Studies, Tamale, Ghana; 5 Ghana Health Service, Dansoman Polyclinic, Dansoman-Accra, Ghana

**Keywords:** Early essential newborn care, Public sector health facilities, Private sector health facilities, Factors, Ghana

## Abstract

**Background:**

Early essential newborn care is one of the important interventions developed by the World Health Organization to reduce morbidities and mortalities in neonates. This study investigated the role of the public and private sector health facilities on factors associated with early essential newborn care practices following institutional delivery in Ghana.

**Methods:**

We used data from the 2017/2018 multiple indicator cluster survey for our analysis. A total of 2749 mothers aged 15–49 years were included in the study. Logistic regression analysis was used to assess the factors associated with early essential newborn care in both public and private health sectors.

**Results:**

The prevalence of good early essential newborn care in the public sector health facilities was 26.4 % (95 % CI: 23.55, 29.30) whiles that of the private sector health facilities was 19.9 % (95 % CI: 13.55, 26.30). Mothers who had a Caesarean section in the public sector health facilities had 67 % lower odds of early essential newborn care compared to mothers who had a vaginal delivery [adjusted prevalence odds ratios (aPOR) = 0.33, 95 % CI: 0.20, 0.53]. Mothers without a health insurance in the public sector health facilities had 26 % lower odds of early essential newborn care compared to mothers with a health insurance (aPOR = 0.74, 95 % CI: 0.56, 0.97). However, these associations were not observed in the private sector health facilities.

**Conclusions:**

The findings suggest that the prevalence of good early essential newborn care in the public sector health facilities was higher than that reported in the private sector health facilities. Child health programs on early essential newborn care needs to be prioritized in the private healthcare sector. The Government of Ghana may also need to increase the coverage of the national health insurance scheme for women in reproductive age.

## Background

Globally, neonatal mortality accounts for nearly half of all deaths in children under five years [1]. Newborns in sub-Saharan Africa (SSA) are ten times more likely to die within the first 28 days of life compared to those born in high income countries [2]. The World Health Organization (WHO) instituted the early essential newborn care (EENC) to reduce neonatal morbidity and mortality. The EENC is an evidence-based intervention that provides quality healthcare to all newborns immediately after birth [3]. The provision of EENC consist of the following: drying of the newborn to reduce hypothermia and stimulate breathing [4, 5]; skin-to-skin contact upon delivery to provide warmth, promote mother-infant bonding, and early initiation of breastfeeding [5–7]; and early initiation of breastfeeding to promote the intake of colostrum by newborns, establish mother and infant bonding, and also promote exclusive breastfeeding [8, 9]. Evidence suggest that adherence to protocols of EENC is associated with reduced neonatal and infant morbidity and mortality [5, 6, 10–12].

Studies have indicated that various factors influence the adherence to EENC practices [7, 13–15]. Demographics such as age and educational level of women have been found to be associated with the practice of EENC [14]. Women aged 35 years or more were less likely to have their babies receive EENC compared to women aged 19 years or less. Longer mother’s years of schooling (11–14 years) was associated with EENC compared to shorter years of schooling (< 7 years). Compliance with EENC practices has been found to be high among trained midwives compared to nurses [15]. Skilled birth attendants who received any form of training on EENC practices had higher compliance to implementation of EENC compared to those who never had any training [5, 16]. Alexsandra et al. found that Caesarean section is negatively associated with EENC practices [14]. Health facilities with adequate equipment and staff are associated with high compliance towards the implementation of EENC practices [7, 16]. Vesel et al. in Ghana also reported that the prevalence of EENC practices were higher in regional and district/private hospitals compared to lower level health facilities such as health centers, clinics or maternity homes [13].

The healthcare system in Ghana is structured under three levels; primary, secondary and tertiary levels of healthcare. While community-based health planning and services (CHPS) compounds, maternity homes, clinics, health centers and district hospitals operate under the primary level of healthcare, regional hospitals and teaching hospitals operate under the secondary and tertiary levels of healthcare respectively [17]. Most of the private sector facilities participate at primary and secondary levels whiles the public sector facilities operates at all the levels of healthcare in Ghana [18]. Although the private sector health facilities constitutes one-third of all health facilities in the country [18], they provide half of all health services in Ghana [19]. The private sector health facilities are also noted for high level of quality care and customer satisfaction when compared to public sector facilities [20]. However, the public sector facilities are the main beneficiaries of interventions aimed at promoting newborn care at the neglect of the private sector facilities. Ghana’s Ministry of Health and its partners usually focus on training health staff in public sector health facilities on newborn care. In addition, logistics and support aimed at promoting newborn care have mostly been channeled to the public sector facilities [21].

There is dearth of knowledge on the prevalence and factors associated with the practice of EENC following institutional deliveries in Ghana. Moreover, studies have not examined the moderating role of the health sector (i.e. private or public) on factors associated with EENC. Our study sought to investigate the role of public and private sector health facilities on factors associated with EENC practices among institutional  deliveries in Ghana.

## Methods

### Study population

The study population was mothers who delivered in a health facility (i.e. institutional delivery) within the past 2 years. Data were analyzed using the 2017–2018 Multiple Indicator Cluster Survey (MICS) conducted in Ghana. The MICS provides robust household data which is nationally representative [22]. The MICS uses a two-stage sampling procedure, which involves the selection of census enumeration areas from each sampling strata whiles systematic random sampling was used to select households from each enumeration area [23].

### Primary outcome

The primary outcome was EENC. EENC was defined as a composite measure using three early essential newborn care practices (early initiation of breastfeeding; skin-to-skin contact; and drying and wrapping of the baby). EENC was categorized as “good early essential newborn care” for newborns that received all three early essential newborn care practices, and “poor early essential newborn care” if a neonate received otherwise. In our study, early initiation of breastfeeding occurred if a mother puts her baby to the breast within one hour of birth [24]. Skin-to-skin contact was defined as putting the baby directly on the bare skin of the mother’s chest immediately after the birth, while drying and wrapping of the baby was defined as drying/wiping and wrapping of the baby immediately after birth.

### Primary variables of interest

The primary variables of interest were skilled attendant at birth (doctor, nurse/midwife); mode of delivery (Caesarean section, vaginal delivery); health facility type (hospital, health centre/clinic, other health facilities); and perceived delivery size of baby at birth (larger than average, average, smaller than average). Mothers who perceived the size of their baby at birth was larger than average, and mothers who perceived their baby was of average size were compared to mothers who perceived their babies were smaller than the average size. Other health facilities refer to maternity homes or CHPS compounds. Health facilities in our study were also categorized into public and private sector facilities. Public sector health facilities refer to health facilities that are government owned whiles private sector health facilities are privately own.

### Covariates

The covariates in our study were mother’s age (15–24, 25–34, 35–49 years); marital status (married/cohabitation, never married); education (no formal education, primary, secondary, college or higher education); and household wealth quintiles (poorest, poor, middle, rich, richest). Other covariates were categorized as place of residence (rural, urban); and parity (0–3, ≥ 4); and having health insurance (yes, no). Parity was defined as number of births (i.e. live and still births) a woman has had, whiles having a health insurance is either public (i.e. national health insurance) or private insurance.

### Data analysis

Descriptive statistics and complex survey multivariable logistic regression models were used to analyze the data. Descriptive statistics were used to characterize our study sample, and to assess the prevalence of good early essential newborn care. We presented the overall prevalence of good early essential newborn care, and also stratified the prevalence by public and private sector facilities using a bar chat.

We assessed the association between the primary variables of interest and primary outcome, while adjusting for covariates. Four separate regression models were conducted. The model one (i.e. univariate model) was an unadjusted model to assess the relationship between all variables (i.e. primary variables and covariates) and our primary outcome. Variables in model one that had a *P*-value < 0.2 [25, 26], or variables that were clinically relevant were included in models two, three, and four. Model two was an adjusted model that assessed the relationship between primary variables of interest and primary outcomes while adjusting for covariates. Models three and four were stratified models of model two that assessed the relationship between the primary variables of interest and outcome among mothers who delivered at facilities in the public and private health sectors respectively. Multicollinearity of the models were assessed using pairwise correlation matrix, variance inflation factor and tolerance, and eigensystem analysis of correlation matrix. Each of the model fitness was assessed using likelihood ratio test. A *P*-value < 0.05 was considered statistically significant.

The complex survey design of MICS was accounted for in all our analyses by applying sample weights, stratification and clustering to ensure the representativeness of our data. We analyzed the data using SAS version 9.3 (SAS Institute, Cary, NC).

## Results

Our study sample comprised of a total of 2749 mothers who delivered in a health facility within the past two years. The number of mothers who delivered in public and private sector health facilities were 2356 and 393 respectively. Majority of the mothers who delivered in public (46.9 %) and private (50.2 %) sector health facilities were aged 25–34 years. While most of mothers in private sector health facilities (63 %) lived in urban area, only 46.5 % of those in the public sector health facilities lived in the urban area. Possession of health insurance was similarly distributed among mothers who delivered in public (66.3 %) and private (68.3 %) sector health facilities. Caesarean section delivery was higher in private sector (20 %) compared to the public sector health facilities (16 %) [Table 1].

**Table 1:** Characteristics of the study population among health facilities in Ghana (*n* = 2749)

Characteristics	Public sector facilities, n (%)	Private sector facilities, n (%)
**Age (years)**		
15–24	690 (29.3)	62 (15.8)
25–34	1105 (46.9)	197 (50.2)
35–49	561 (23.8)	134 (34)
**Marital status**		
Never married	417 (17.7)	44 (11.3)
Married/cohabitation	1939 (82.3)	349 (88.7)
**Education**		
No formal education	476 (20.2)	39 (10)
Primary	466 (19.8)	72 (18.4)
Secondary	1276 (54.2)	227 (57.8)
College or higher education	137 (5.8)	54 (13.8)
**Household wealth**		
Poorest	436 (18.5)	37 (9.4)
Poor	445 (18.9)	53 (13.5)
Middle income	470 (19.9)	54 (13.8)
Rich	521 (22.1)	103 (26.1)
Richest	483 (20.5)	146 (37.2)
**Place of residence**		
Urban	1095 (46.5)	248 (63)
Rural	1261 (53.5)	145 (37)
**Parity**		
0–3	1473 (62.5)	216 (55)
≥ 4	882 (37.5)	177 (45)
**Health insurance**		
Yes	1562 (66.3)	269 (68.3)
No	793 (33.7)	125 (31.7)
**Perceived delivery size of baby**		
Smaller than Average	408 (17.4)	51 (13)
Average	836 (35.6)	146 (37.2)
Larger than Average	1103 (47)	195 (49.8)
**Mode of delivery**		
Vaginal delivery	1978 (84)	315 (80)
Cesarean section	377 (16)	78 (20)
**Health facility type**		
Hospital	1530 (64.9)	292 (74.3)
Health centre/clinic	740 (31.4)	75 (19.2)
Others	86 (3.7)	26 (6.6)
**Skilled attendant**		
Doctor	200 (8.5)	42 (10.8)
Nurse/Midwife	2146 (91.5)	346 (89.2)

**Fig. 1:**
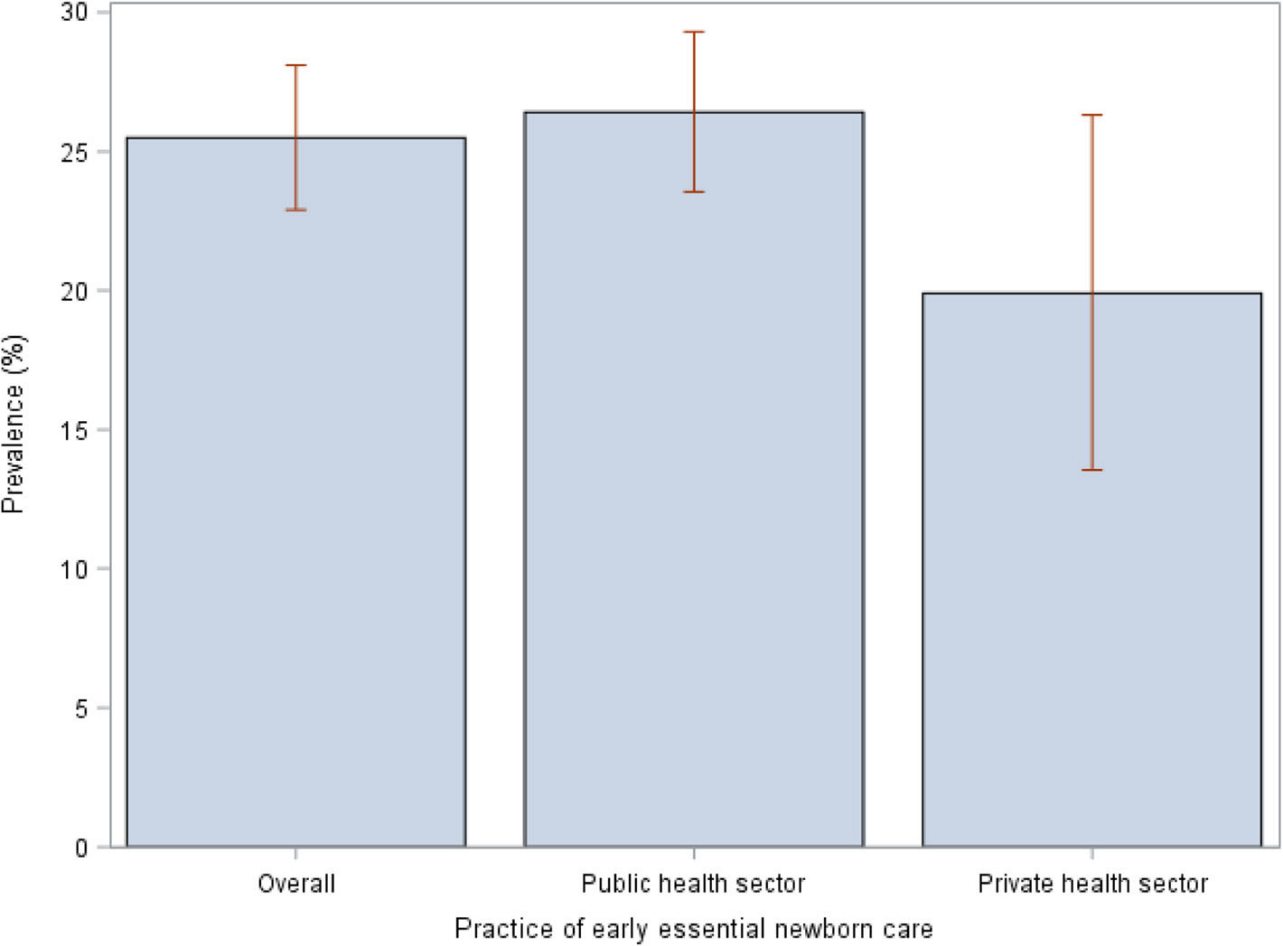
Practice of early essential newborn care, overall and stratified by health sector

An estimated 25.5 % (95 % CI: 22.90, 28.09) of the newborns received good EENC (i.e. newborns who received drying and wrapping, skin-to-skin contact and early initiation of breastfeeding). The prevalence of drying and wrapping, skin-to-skin and early initiation of breastfeeding were 38.3 %, (95 % CI: 35.80, 40.80), 64.4 % (95 % CI: 61.80, 67.00) and 52.3 % (95 % CI: 49.70, 54.90) respectively (results not shown). The prevalence of newborns who received good EENC in public and private sector facilities were 26.4 % (95 % CI: 23.55, 29.30) and 19.9 % (95 % CI: 13.55, 26.30), respectively [Fig. 1].

The multivariable logistic regression revealed that newborns who were delivered by caesarean section received 66 % lower odds of good EENC compared to mothers who had vaginal delivery. Mothers without health insurance had lower odds of good EENC for their newborns compared to mothers who had health insurance [Adjusted prevalence odds ratio (aPOR): 0.72, 95 % CI: 0.56, 0.93]. Among mothers who delivered in public sector health facilities, newborns who were delivered by caesarean section (aPOR = 0.33, 95 % CI: 0.20, 0.53), and whose mothers had no health insurance had lower odds of good EENC (aPOR = 0.74, 95 % CI: 0.56, 0.97), compared to newborns who were delivered by vaginal delivery, and whose mothers had a health insurance respectively. Among mothers who delivered in private sector health facilities, none of the variables was associated with the outcome [Table 2].

**Table 2:** Factors associated with early essential newborn care during institutional delivery and stratified by public and private sectors health

Characteristics	Model 1 Unadjusted OR (95 % CI)	Model 2 Adjusted OR (95 % CI)	Model 3 Adjusted OR (95 % CI)	Model 4 Adjusted OR (95 % CI)
**Age (years)**				
15–24	1	1	1	1
25–34	0.95 (0.71,1.26)	0.97 (0.69,1.37)	0.94 (0.65,1.35)	1.12 (0.30,4.12)
35–49	1.02 (0.75,1.38)	1.06 (0.68,1.66)	1.21 (0.76,1.92)	0.43 (0.08,2.17)
**Marital status**				
Never married	1	1	1	1
Married/cohabitation	1.11 (0.81,1.51)	1.14 (0.79,1.65)	1.24 (0.84,1.82)	0.63 (0.19,2.12)
**Education**				
No formal education	1	1	1	1
Primary	0.86 (0.56,1.32)	0.96 (0.62,1.50)	0.96 (0.59,1.55)	0.82 (0.17,3.97)
Secondary	0.84 (0.61,1.16)	0.96 (0.66,1.39)	1.04 (0.70,1.56)	0.41 (0.12,1.47)
College or higher education	0.67 (0.39,1.15)	0.88 (0.48,1.63)	0.86 (0.42,1.76)	0.65 (0.16,2.66)
**Household wealth**				
Poorest	1	1	1	1
Poor	1.06 (0.76,1.47)	1.11 (0.78,1.57)	1.15 (0.79,1.67)	0.75 (0.14,4.07)
Middle income	0.97 (0.66,1.41)	1.04 (0.69,1.57)	1.06 (0.69,1.63)	0.69 (0.11,4.31)
Rich	0.75 (0.52,1.08)	0.90 (0.59,1.37)	0.88 (0.56,1.40)	1.41 (0.23,8.63)
Richest	0.89 (0.60,1.33)	1.19 (0.70,2.01)	1.13 (0.65,1.94)	2.34 (0.37,14.67)
**Place of residence**				
Urban	1	1	1	1
Rural	1.29 (0.98,1.70)	1.17 (0.86,1.60)	1.14 (0.81,1.59)	1.83 (0.57,5.83)
**Parity**				
0–3	1	1	1	1
≥ 4	1.05 (0.82,1.36)	0.97 (0.69,1.36)	0.98 (0.68,1.40)	1.03 (0.38,2.75)
**Health insurance**				
Yes	1	1	1	1
No	0.76 (0.60,0.96)	0.72 (0.56,0.93) *	0.74 (0.56,0.97) *	0.56 (0.23,1.41)
**Perceived delivery size of baby**				
Smaller than Average	1	1	1	1
Average	1.18 (0.82,1.68)	1.09 (0.77,1.54)	1.09 (0.76,1.56)	1.41 (0.39,5.06)
Larger than Average	1.35 (0.96,1.89)	1.26 (0.90,1.75)	1.30 (0.90,1.86)	1.15 (0.36,3.75)
**Mode of delivery**				
Vaginal delivery	1	1	1	1
Caesarean section	0.32 (0.22,0.48)	0.34 (0.22,0.52) *	0.33 (0.2,0.53) *	0.49 (0.15,1.61)
**Health facility type**				
Hospital	1	1	1	1
Health centre/clinic	1.29 (0.98,1.7)	1.06 (0.79,1.42)	1.09 (0.80,1.46)	0.67 (0.19,2.38)
Others	1.41 (0.69,2.89)	1.30 (0.63,2.65)	1.44 (0.66,3.15)	0.59 (0.10,3.47)
**Skilled attendant**				
Doctor	1	1	1	1
Nurse/Midwife	2.03 (1.28,3.22)	1.31 (0.78,2.19)	1.22 (0.70,2.13)	3.49 (0.57,21.32)

*P-value less than 0.05

## Discussion

This study investigated the role of public and private sector health facilities on factors associated with EENC practices following institutional  deliveries in Ghana. About one-quarter of babies born in public sector health facilities received good EENC practices whiles an estimated one in five children born in the private sector health facilities received good EENC practices. Caesarean delivery and not having health insurance were associated with lower odds of good EENC practices in the public sector health facilities. However, we did not find any association with the primary outcome in the private sector health facilities.

We found that good EENC practices in the public sector health facilities was higher than those of the private sector health facilities. Several reasons could account for this difference. Firstly, the public sector health facilities has a higher number of skilled health professionals compared to the private sector health facilities [27], and skilled health professionals are more likely to adhere to good EENC practices [28]. In addition, in-service trainings on newborn care from the Ministry of Health and its partners have mostly benefited staff from the public sector health facilities [21]. There is also higher participation in continuous professional development training among staff in the public sector health facilities compared to the private sector health facilities; and knowledge of EENC has been found to be associated with compliance with good EENC practices [5, 16].

Our study also found that Caesarean section was associated with lower odds of good EENC practices in the public sector health facilities. A number of possible reasons might account for this observation. Mothers who delivered by Caesarean section may be uncomfortable adopting to good EENC practices such as early initiation of breastfeeding as they may need some time to recover from anaesthesia [9, 29]. Furthermore, newborns who were delivered by Caesarean section might suffer birth asphyxia requiring resuscitation that may lead to separation from the mother hence skin to skin and early initiation of breastfeeding will not immediately be possible [9]. Nevertheless, good EENC such as early skin-to-skin contact initiated post Caesarean section has not been found to be associated with any significant risks with regard to neonatal well-being. In this vein, it is essential to commence good EENC practices as early as possible following Caesarean section delivery [30]. Cognizant of the increasing Caesarean section rates globally and in Ghana, it is also imperative that institutional Caesarean section rates be maintained within optimal range so as to improve of neonatal health [31–33]. The results of this study also showed that not having health insurance was associated with lower odds of good EENC practices compared to their peers with a health insurance. Mothers without a health insurance have lower odds of institutional delivery [34, 35]. Since our study was conducted in health facilities, it is likely that fewer mothers without health insurance participated in the study, and that might account for this finding. The National Health Insurance Scheme (NHIS) in Ghana is very useful as it covers maternal health services including prenatal care, delivery care, postnatal care, and healthcare for child up to 3 months post-delivery. Educating pregnant women on its advantages and encouraging them to take up NHIS will expand coverage, increase institutional delivery, and improve good EENC practices [36].

Although Caesarean section and not having health insurance were observed to be associated with poor EENC practices in the public sector health facilities, same was not observed in the private sector health facilities. Several reasons may possibly explain these findings. With regard to caesarean section, this finding might be due the fact that women who deliver at private health facilities in Ghana are more likely to undergo Caesarean section [37], and that might be the reason for our observation. Furthermore, some private sector health facilities levy clients for user fees irrespective of their health insurance status [38], therefore health insurance status may not play an important role in good EENC practices. The null findings of the association between caesarean section, health insurance and good EENC practices could also be due to random error.

The study had some strengths and limitations. Our study was representative and our findings can therefore be generalized in Ghana. Causal relationships cannot be established due to the cross-sectional design of the study. Most of the variables were self-reported and may be affected by recall bias, however, we expect recall bias to be similar between mothers who reported good EENC and those that did not. Moreover, perceived size of the baby at delivery was self-reported and therefore subject to measurement error as this may not be a reflection of the actual weight of the baby at delivery. However, we also expect measurement error to be similar among neonates who received good EENC and those that did not.

## Conclusions

The prevalence of good EENC practices was higher in the public sector health facilities compared to the private sector health facilities. Caesarean section and no health insurance were associated with lower odds of good EENC practices in the public sector health facilities. However, same was not observed with the private sector health facilities. Efforts to improve good EENC practices in the public sector health facilities should target women who deliver by caesarean section and also those with no health insurance towards the attainment of optimal neonatal health.

## Data Availability

MICS data is publicly available at: https://mics.unicef.org/surveys.
